# East Texas Medical Society

**Published:** 1896-08

**Authors:** E. A. Woldert

**Affiliations:** Secratary


**The East Texas Medical Association** held its regular quarterly meeting in Tyler, on the 14th and 15th of July, ult., with a good attendance, and added eight new members. An election was held and the officers for the ensuing year are as follows:

Dr. T. J. Bell, president, Tyler; Dr. A. M. Denman, vice-president, Lufkin; Dr. H. O. Lyford, second vice-president, Jacksonvile; Dr. E. A. Woldert, secretary, Tyler; Dr. C. A. Smith, treasurer, Tyler.

Place of next meeting Jacksonville, Texas, second Tuesday in January.

E. A. WOLDERT, Secratary.

